# Changes in CMR-derived ventricular strain, fibrosis progression and outcomes in hypertrophic cardiomyopathy

**DOI:** 10.1007/s10554-026-03636-6

**Published:** 2026-01-27

**Authors:** Alberto Aimo, Andrea Barison, Annamaria Del Franco, Chrysanthos Grigoratos, Alessandro Parlato, Chiara Zocchi, Giovanni Donato Aquaro, Giorgia Panichella, Carmelo De Gori, Ignazio Gueli, Alessandro Folgheraiter, Antonio De Luca, Marco Merlo, Michele Emdin, Gianfranco Sinagra, Iacopo Olivotto, Giancarlo Todiere

**Affiliations:** 1 Interdisciplinary Center for Health Sciences, Scuola Superiore Sant’Anna, Pisa, Italy; 2 Cardiology Division, Fondazione Toscana Gabriele Monasterio, Pisa, Italy; 3 Cardiomyopathy Unit, Careggi University Hospital, Florence, Italy; 4 Cardiology Division, University Hospital of Pisa, Pisa, Italy; 5 Academic Radiology Unit, Department of Surgical Medical and Molecular Pathology and Critical Area, University of Pisa, Pisa, Italy; 6 Department of Experimental and Clinical Medicine, University of Florence, Florence, Italy; 7 Radiology Division, Fondazione Toscana Gabriele Monasterio, Pisa, Italy; 8 Cardiology Division, University Hospital of Trieste, Trieste, Italy; 9 Cardiology Unit, Meyer Children’s Hospital IRCCS, Florence, Italy; 10 Scuola Superiore Sant’Anna and Fondazione Toscana Gabriele Monasterio, Pisa, Italy

**Keywords:** Hypertrophic cardiomyopathy, HCM, Cardiac magnetic resonance, Strain, Risk prediction

## Abstract

Hypertrophic cardiomyopathy (HCM) is a major health concern, with cardiac magnetic resonance (CMR) playing a crucial role in risk assessment. We investigated for the first time the utility of sequential CMR, particularly strain analysis, for tracking HCM progression. We retrospectively evaluated HCM patients undergoing two CMR scans over a 10-year period. We measured changes in left ventricular (LV) strain parameters and examined their yearly changes as predictors of a composite of sudden cardiac death, life-threatening ventricular arrhythmias, stroke, new-onset atrial fibrillation, and heart failure hospitalizations. Patients (*n* = 114) were predominantly male (73%), with a median age of 51 years (interquartile range 36–60), obstructive HCM in 14%, and a median HCM risk score of 2% (1–3%). Only one patient (0.9%) had LGE ≥ 15% on the first scan, while 8 (7.0%) had LGE ≥ 15% after a median of 5.1 years (3.5–6.5). Absolute changes in LV strain displayed significant relationships with changes in LGE mass (longitudinal strain: beta = 0.227, *p* = 0.016; circumferential strain: beta = 0.421, *p* < 0.001; radial long-axis: beta=-0.261, *p* = 0.006). During a 4.3-year median follow-up after the second CMR scan (interquartile range 2.2–6.9), 40 patients experienced an event; hard arrhythmic events were infrequent. Among patients with LGE < 15% at baseline, yearly absolute changes in radial short-axis strain predicted outcomes beyond baseline HCM score and LGE extent (hazard ratio 1.12, 95% confidence interval 1.03–1.22, *p* = 0.011). In patients with predominantly early-stage HCM, worsening short-axis radial strain was associated with composite adverse events after the second CMR, independent of baseline LGE and ESC SCD risk.

**Supplementary Information:**

The online version contains supplementary material available at 10.1007/s10554-026-03636-6.

## Background

Hypertrophic cardiomyopathy (HCM) is characterized by increased left ventricular (LV) wall thickness or mass not fully explained by abnormal loading conditions [1]. It is a leading cause of sudden cardiac death in young individuals, with a broad clinical spectrum that spans atrial fibrillation (AFib), malignant ventricular arrhythmias, and dilated forms [2]. Although some patients advance rapidly to severe disease, HCM typically follows a slow remodeling course with low event rates, often remaining asymptomatic for decades.

Cardiovascular magnetic resonance (CMR) is ideally suited to characterize early, subtle changes in LV function and myocardial fibrosis due to its non-invasive nature, independence from acoustic windows, and comprehensive tissue assessment [3]. Current guidelines recommend performing CMR at the initial evaluation (class I, level of evidence B) and periodically thereafter (every 2–5 years, class IIa, level of evidence C) to monitor disease progression and refine risk stratification [1]. By accurately visualizing areas of LV hypertrophy often missed on echocardiography, quantifying wall thickness, and differentiating HCM from other causes of hypertrophy, CMR highlights specific subsets of patients (e.g., those with apical aneurysms or LV outflow obstruction) [3]. Moreover, late gadolinium enhancement (LGE) identifies and quantifies myocardial fibrosis—a major risk factor for life-threatening arrhythmias and a potential indication for defibrillator implantation [4]. Even modest increases in LV fibrosis may prove significant over time, making serial LGE assessment particularly valuable for elucidating gradual remodeling.

Beyond fibrosis imaging, global strain analysis derived from CMR feature tracking offers a sensitive measure of early myocardial involvement, including in genotype-positive, phenotype-negative individuals [5]. CMR-derived global longitudinal strain is also an independent predictor of major adverse cardiac events, beyond established risk factors such as LV ejection fraction (LVEF) and LGE burden [6]. Strain impairment and fibrosis progression likely stem from excessive energetic demands, microvascular ischemia, and hypercontractile sarcomere function in HCM. Identifying small yet clinically meaningful changes in strain and fibrosis is increasingly important as emerging treatments have the potential to modify the disease course in its earlier stages.

In this study, we evaluated HCM patients with predominantly mild fibrosis and largely non-obstructive disease, evaluated at baseline and after a median of 5 years, to: (1) determine the mean annual rate of LGE progression (including transitions above the critical 15% threshold), (2) investigate the relationship between changes in LGE extent and LV strain parameters, and (3) explore how these shifts in myocardial fibrosis and strain may influence subsequent clinical outcomes.

## Methods

### Patient population

We screened consecutive patients with HCM who underwent clinical CMR at three tertiary laboratories between 2014 and 2024 (Fondazione Toscana G. Monasterio, Pisa/Massa; Careggi University Hospital, Florence; University Hospital of Trieste). We identified all individuals with ≥ 2 analyzable CMR examinations. In routine practice at these centers, and in line with the 2023 ESC cardiomyopathy guidelines [1], patients with HCM are advised to undergo follow-up CMR every 3–5 years for surveillance. Approximately one-third of patients with an index CMR did not undergo a second CMR within 5 years. Documented reasons included clinical stability with echocardiographic surveillance alone, device-related limitations or institutional policy, transfer of care/relocation, patient preference or claustrophobia, intercurrent illness or death, renal dysfunction/contrast avoidance, and post-procedural changes (e.g., septal reduction therapy).

Among patients with two CMR scans available, patients were excluded if either CMR lacked complete cine coverage (short-axis stack plus 2-, 3-, and 4-chamber views) and LGE imaging, or if image quality/gating artifacts precluded reliable feature-tracking strain or LGE quantification at either time point. Additional exclusions were secondary/alternative cardiomyopathies or septal reduction therapy between the two scans. The final cohort comprised 114 patients, in whom all contours and strain/LGE analyses were re-performed centrally using the same cvi42 version to ensure consistency across time points and centers. A detailed flow diagram of screening, exclusions, and inclusion is provided in Supplemental Fig. 1.

HCM was diagnosed and managed according to contemporary guidelines [1, 7]. Obstructive HCM was defined from the echocardiogram closest to the first CMR as an LV outflow tract gradient ≥ 30 mmHg at rest or ≥ 50 mmHg with provocation (Valsalva and/or exercise); the highest value determined classification [1, 7]. Informed consent was obtained from each patient. The study protocol conforms to the ethical guidelines of the 1975 Declaration of Helsinki as reflected by a priori approval by the institutions’ human research committees. No funding was received.

### Cardiovascular magnetic resonance protocol

Patients were examined by 1.5-T CMR systems (FTGM: Signa CVi and Signa Artist, GE-Healthcare, Milwaukee, USA; Careggi Hospital: MAGNETOM Aera, Siemens Healthineers, Erlangen, Germany; University Hospital of Trieste: Philips Achieva and Philips Ingenia) using a phased-array surface receiver coil and vectorcardiogram triggering. Cine imaging used breath-hold balanced SSFP in contiguous short-axis stacks covering the entire LV from base to apex. All the scans were performed using a 8-mm slice thickness with no gap, which is the standard short axis cine and LGE protocol in use in all the three CMR centers, to increase image quality and cover all the myocardium. For the analysis, all contours were redone *de novo* at the core lab using the same cvi42 version to minimize software/version effects. Typical cine parameters were: field-of-view 380–400 mm, TR/TE ~ 3.2/1.6 ms, flip angle ~ 60°, matrix ~ 224 × 192. All cine images were acquired using 30 cardiac phases in all three centers, with an average temporal resolution set to 40–60 ms according to patients’ heart rate and breath holding capacity. Patients with poor image quality due to breathing or arrhythmia artifacts were excluded from analysis.

LGE imaging used inversion-recovery gradient-echo sequences 10–20 min after contrast, with inversion time set to null remote myocardium. Short-axis LGE was acquired with contiguous 8–10 mm slices and no inter-slice gap whenever feasible; LGE presence/extent was confirmed in 2- and 4-chamber views. Typical parameters: field-of-view 380–400 mm, TR/TE ~ 4.6/1.3 ms, matrix ~ 256 × 192.

**Fig. 1:**
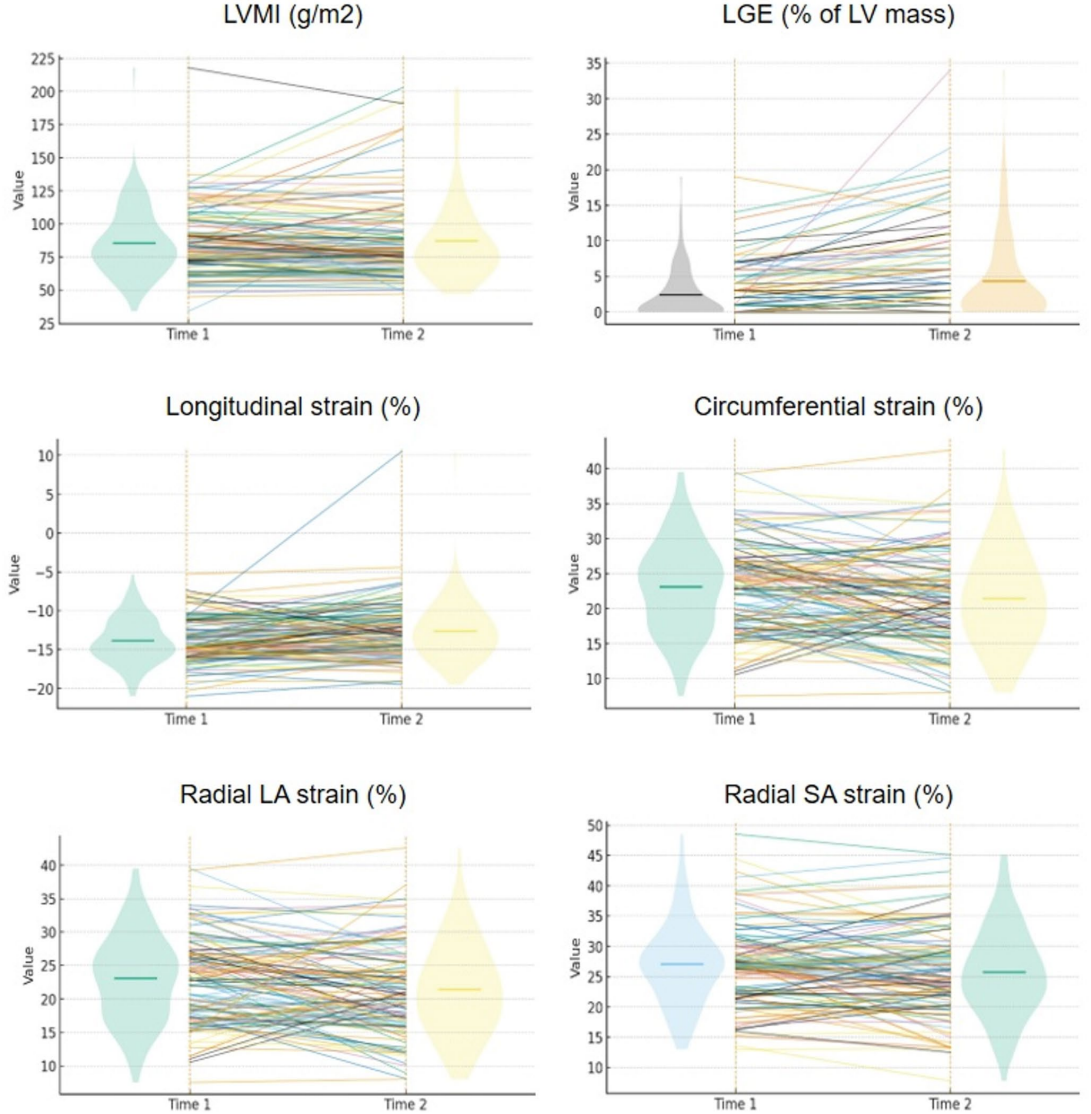
Changes in cardiovascular magnetic resonance (CMR)-derived metrics in hypertrophic cardiomyopathy (HCM). Spaghetti plots with side violins showing paired changes between time 1 (left) and time 2 (right) for six metrics. Each thin line traces one participant’s values across the two times; jittered dots are the individual observations. Semi-transparent violins summarize the distribution at each time point (width proportional to density) and the horizontal tick marks the mean. Panels depict: left ventricular mass index (LVMI), late gadolinium enhancement (LGE), longitudinal strain, circumferential strain, radial long-axis (LA) and short-axis (SA) strain. p values for comparisons: LVMI, *p* = 0.451; LGE: *p* < 0.001; longitudinal strain, *p* < 0.001; circumferential strain, *p* = 0.020; radial LA strain, *p* = 0.002; radial SA strain, *p* = 0.053

Across centers and years, macrocyclic gadolinium-based agents were used according to local formularies. The default agent at Pisa was gadoteric acid (Dotarem) at 0.2 mmol/kg; at the other sites macrocyclic agents of comparable relaxivity (e.g., gadobutrol or gadoteridol) were also used when Dotarem was not available. Because LGE was quantified with a relative signal-intensity threshold (6-SD above remote myocardium) and standardized timing post-injection, analyses are not expected to be materially affected by the specific macrocyclic agent used (Fig. 1).

### Image analysis

**Fig. 2:**
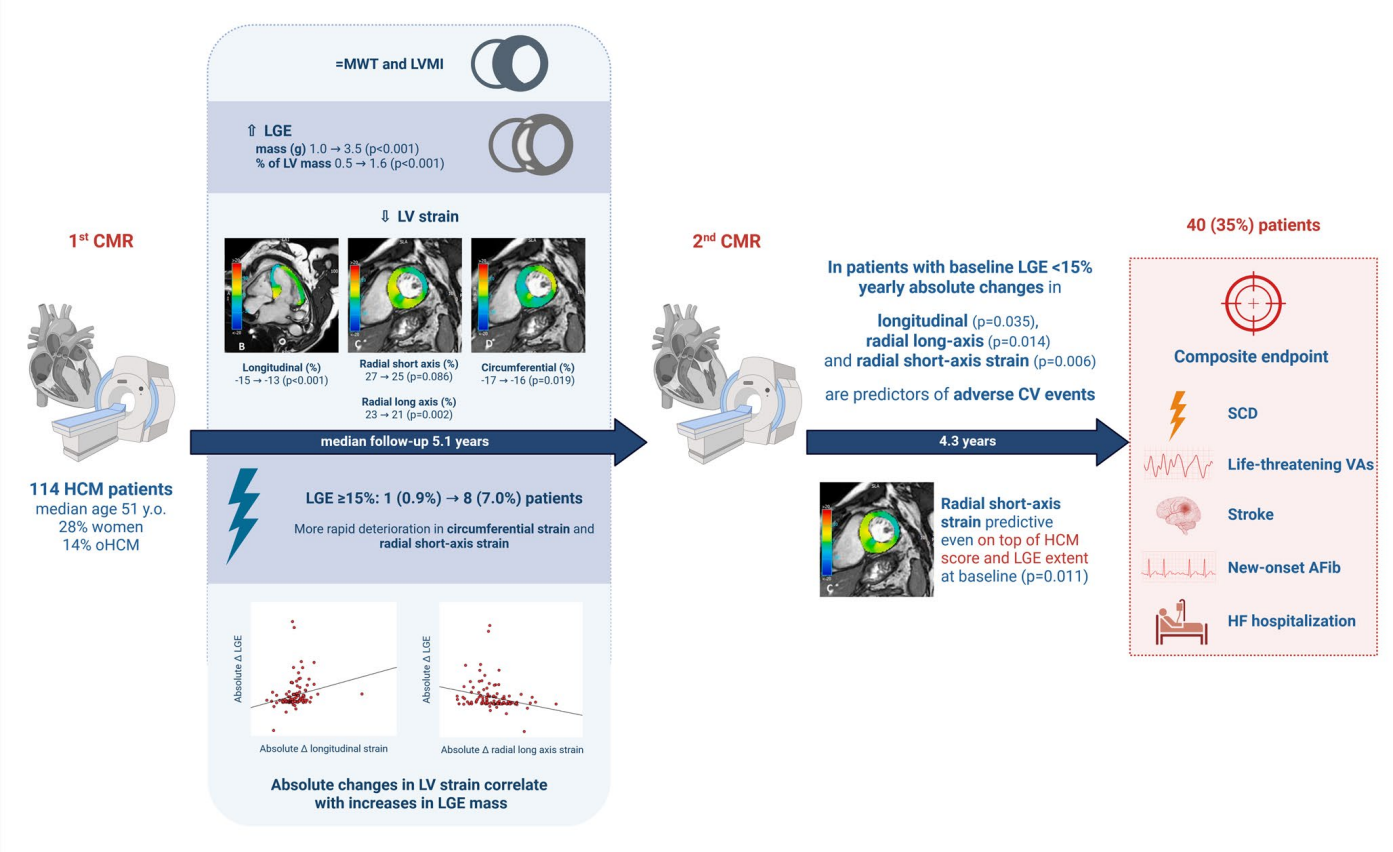
Central Illustration. Changes in left ventricular (LV) strain: relationship with increase in late gadolinium enhancement (LGE) and outcome. See text for details. AFib, atrial fibrillation; CMR, cardiovascular magnetic resonance; HCM, hypertrophic cardiomyopathy; HF, heart failure; LVMI, left ventricular mass index; MWT, mean wall thickness; oHCM, obstructive hypertrophic cardiomyopathy; VA, ventricular arrhythmia

In patients with atrial fibrillation or frequent ectopy, we used arrhythmia-rejection when available and repeated segmented acquisitions to obtain a representative cycle; when necessary for selected views, real-time cine was acquired to aid border delineation. During post-processing, end-diastole and end-systole were identified visually on long- and short-axis views; feature-tracking was performed on cine series with adequate gating and without ectopic beats, and segments with mistracking were re-initialized or excluded according to predefined quality-control rules. All analyses were performed centrally by experienced readers blinded to time point and outcomes using cvi42 version 5.13.7 (Circle Cardiovascular Imaging Inc., Calgary, AB, Canada). Using the stack of short-axis cine images, LV and right ventricular volumes, mass and global function were calculated [8]. LV mean wall thickness was determined by direct measurement at end-diastole of the thickest LV segment. In all 17 segments (according the American Heart Association/American College of Cardiology classification), LGE was graded visually, both in location (midwall, subepicardial, subendocardial-to-transmural) and transmural extent (as quartiles of the wall thickness); patients with only faint LGE areas limited to the right ventricular insertion points were considered LGE-negative. Hyperenhanced areas (with a signal intensity > 6 standard deviations above remote normal myocardium) in the LV myocardium were manually adjusted and were summed over the entire LV myocardium to calculate scar mass as a percentage of the LV mass (Fig. 2).

Left atrial volumes in atrial systole and diastole were calculated using the following formula:

$$\:LAV=\frac{8*A4C*A2C}{3*\pi\:*L}$$

where A4C and A2C are the left atrial areas in 4- and 2-chamber acquisitions and L is the shortest atrial length measured between 2- and 4-chambers [9]. Left atrial area measurements excluded atrial appendage and mitral tenting area. Left atrial EF was then calculated through the standard EF formula.

Strain analysis was performed retrospectively by experienced CMR readers blinded to scan timepoints and outcomes using the cvi42^®^ software via post-processing feature tracking. End-systole and end-diastole phases were chosen manually by visually inspecting ventricular dimensions and valves movements in long and short axis views. Simplified endocardial and epicardial borders were then automatically derived by the software protocols and manually adjusted when needed. In short-axis acquisitions, only slices where complete endocardial and epicardial rings could be traced were included [10]. LV global longitudinal, circumferential and radial strain values were then derived. Inter-operator reproducibility of strain parameters was assessed on a random sample of 10 CMR exams evaluated independently by two expert CMR readers. Intra-operator reproducibility was assessed by repeating the strain analysis after at least 30 days, blinded to the results of the first analysis.

### Follow-up

Follow-up data were collected from the electronic health records and phone calls to the patients and their relatives. The primary endpoint was a composite of sudden cardiac death (SCD), ventricular fibrillation or hemodynamically unstable ventricular tachycardia, stroke, new-onset AFib or heart failure (HF) hospitalization.

### Statistical analysis

The IBM SPSS Statistics v24 and Python 3.11 software were used. Continuous variables were assessed for normality with the Shapiro–Wilk test and are presented as median and interquartile range (IQR) given the predominantly non-normal distributions; categorical variables are reported as counts and percentages. Between-group comparisons used the χ² test or Fisher’s exact test (when expected counts < 5) for categorical data, and the Mann–Whitney U test for continuous data. Within-patient changes between the first and second CMR were assessed with the Wilcoxon signed-rank test. Inter- and intra-observer reproducibility of strain measurements was evaluated with two-way random-effects, absolute-agreement intraclass correlation coefficients (ICC), with ICC > 0.80 considered acceptable. Associations between changes in strain and CMR metrics were explored with Spearman rank correlation. To report effect sizes consistently with tables, simple linear regression models were additionally fitted to obtain standardized beta coefficients for change–change relationships. Yearly absolute change in strain was defined as value at second CMR minus value at first CMR divided by the inter-scan interval (years). Prognostic analyses followed a landmark approach with time zero at the second CMR. Annualized strain change was computed from the first and second CMR examinations (pre-landmark) and entered as a fixed baseline covariate; covariates were not updated during follow-up. Univariable Cox regression models evaluated each strain change; multivariable models adjusted for the European Society of Cardiology (ESC) HCM risk score and baseline LGE extent (% LV mass). The primary prognostic analyses were prespecified in the subgroup with baseline LGE < 15% of LV mass. Proportional-hazards assumptions were checked with Schoenfeld residuals and log–log plots. For visualization, the relationship between yearly absolute changes and the endpoint was depicted with restricted cubic spline terms (natural splines) embedded in Cox models; spline flexibility (degrees of freedom 3–8) was tuned using the Bayesian Information Criterion, and curves were plotted over the observed range of each predictor. Two-sided p-values < 0.05 were considered statistically significant. Complete-case analyses were used throughout.

## Results

### Patient characteristics and CMR findings

The study cohort included 114 HCM patients, with 27% women. 40% had a genetic variant deemed pathogenetic. At the time of the first CMR scan, median age was 51 years (interquartile range 36–60), and the median HCM risk score was 2% (1–3%); 16 patients (14%) had an HCM risk score ≥ 4%, and 7 (6%) had a risk ≥ 6% (Table 1). Sixteen patients (14%) had obstructive HCM. Compared to patients with non-obstructive HCM, they were older and had more often dyspnea on effort, while their HCM risk score did not differ significantly (Supplemental Table 1).

**Table 1:** Patient characteristics

	Patients *n* = 114
Men, n (%)	83 (73)
Age (years)	51 (36–60)
Palpitations, n (%)	35 (31)
Unexplained syncope, n (%)	6 (5)
Angina, n (%)	25 (22)
Dyspnea on effort, n (%)	36 (32)
Atrial fibrillation, n (%)	19 (17)
NSVT, n (%)*	14 (17)
LVOT gradient (mmHg)	5 (2–12)
Obstructive HCM, n (%)	16 (14)
Maximal LV wall thickness (mm)	17 (15–20)
Left atrial diameter (mm)	38 (34–43)
Family history of SCD, n (%)*	12 (15)
HCM score (%)	2 (1–3)

* Percentage calculated out of the available data. HCM, hypertrophic cardiomyopathy; LV, left ventricle; LVOT, left ventricular outflow tract; NSVT, non-sustained ventricular tachycardia; NYHA, New York Heart Association; SCD, sudden cardiac death

### First vs. second CMR scan

No patient underwent ICD implantation after the first CMR scan. The second CMR scan was performed after a median interval of 5.1 years (IQR 3.5–6.5) from the first scan. Fifty patients (44%) displayed any increase in LV mass index (LVMI), but overall LVMI did not change significantly. Conversely, LGE mass increased from 1.0 g (0.1–5.7) to 3.5 g (0.8–11.5; *p* < 0.001), and LGE extent from 0.5% of LV mass (0.1–3.6) to 1.6% (0.5–6.1; *p* < 0.001) (Table 2; Fig. 1). The rate of increase in LGE mass was 0.2 g/year (0–0.9 g/year), and the rate of increase in LGE extent was 0.1% points per year (0–0.5.5%).

**Table 2:** Findings from cardiovascular magnetic resonance (CMR) scans

	1 st CMR *n* = 114	2nd CMR *n* = 114	*p*
LV EDVi (mL/m^2^)	76 (67–91)	78 (67–89)	0.846
LV ESVi (mL/m^2^)	27 (19–32)	27 (21–33)	0.726
LV EF (%)	67 (62–72)	65 (60–71)	0.808
Max LV wall thickness (mm)	17 (15–20)	18 (15–20)	0.102
LVMI (g/m^2^)	81 (70–101)	79 (66–97)	0.456
LGE mass (g)	1.0 (0.1–5.7)	3.5 (0.8–11.5)	**< 0.001**
LGE extent (% of LV mass)	0.5 (0.1–3.6)	1.6 (0.5–6.1)	**< 0.001**
LGE ≥ 15%, n (%)	1 (0.9)	8 (7.0)	**< 0.001**
Left atrial max volume (mL)	107 (84–137)	105 (84–151)	0.086
Left atrial EF (%)	50 (42–56)	46 (35–52)	**< 0.001**
LV strain			
Longitudinal (%)	−15 (−16/−11)	−13 (−15/−11)	**< 0.001**
Circumferential (%)	−17 (−18/−15)	−16 (−18/−14)	**0.019**
Radial long-axis (%)	23 (18–27)	21 (16–26)	**0.002**
Radial short-axis (%)	27 (22–31)	25 (21–30)	0.080

Significant p values are reported in bold. EDVi, end-diastolic volume index; EF, ejection fraction; ESVi, end-systolic volume index; LGE, late gadolinium enhancement; LV, left ventricle; LVMI, left ventricular mass index

Only one patient (0.9%) had LGE ≥ 15% on the first scan, but 8 patients (7.0%) progressed from LGE < 15% to LGE ≥ 15%. Patients progressing to LGE ≥ 15% had a much higher annual rate of LGE increase than those with LGE still < 15%: 2.2 g/year (1.6–6.1) vs. 0.1 g/year (0–0.7.7; *p* < 0.001), and 1.4% (1.0–4.4.0.4) vs. 0.1% (0–0.4.4; *p* < 0.001), respectively.

### Strain analysis: values, correlates and changes

Intra-operator reproducibility of strain analysis largely exceeded the established cut-off of 0.8 with average ICC of 0.97 for global longitudinal strain, 0.94 for circumferential strain, and 0.93 for radial strain. Similar results were obtained for inter-operator variability (0.95, 0.97, and 0.95, respectively).

Longitudinal, circumferential and radial long-axis strain values deteriorated significantly from the first to the second CMR scan (Table 2; Fig. 1 and Supplemental Table 2). Changes in strain values correlated with increases in LGE mass and extent (Table 3). Therefore, strain values deteriorated in parallel with increasing amounts of fibrosis. Patients progressing from LGE < 15% to ≥ 15% displayed a more rapid deterioration in circumferential strain and radial short-axis strain (Table 4).

**Table 3:** Correlations between absolute changes in strain values and left ventricular (LV) mass and fibrosis

	Max LV wall thickness	LVMI	LGE mass	LGE extent
**Longitudinal**	*p* = 0.003 Beta=−0.278	*p* = 0.005 Beta = 0.264	*p* = 0.016 Beta = 0.227	*p* = 0.050
**Circumferential**	*p* = 0.006 Beta = 0.256	*p* = 0.073	*p* < 0.001 Beta = 0.412	*p* < 0.001 Beta = 0.455
**Radial long-axis**	*p* < 0.001 Beta=−0.364	*p* < 0.001 Beta=−0.272	*p* = 0.006 Beta=−0.261	*p* = 0.030 Beta=−0.206
**Radial short-axis**	*p* = 0.244	*p* = 0.458	*p* = 0.058	*p* = 0.039 Beta=−0.196

Significant p values were reported in bold. LGE, late gadolinium enhancement; LVMI, left ventricular mass index

**Table 4:** Absolute changes in strain values in patients progressing to late gadolinium enhnacement (LGE) ≥ 15%

	LGE < 15% first scan, ≥15% second scan *n* = 8	LGE ≥ 15% first scan or nor progressing to LGE ≥ 15% *n* = 8	*p*
**Longitudinal**	+ 1.6 (+ 0.6 to + 2.7)	+ 1.2 (−0.6 to + 2.9)	0.538
**Circumferential**	+ 2.9 (+ 1.8 to + 5.8)	0 (−1.2 to + 1.6)	**0.001**
**Radial long-axis**	−3.8 (−5.2 to −1.7)	−1.5 (−6.0 to + 2.4)	0.274
**Radial short-axis**	−6.9 (−13.1 to −4.1)	0.1 (−3.4 to + 2.9)	**0.001**

Significant p values were reported in bold

### Patient outcomes

Over a median of 4.3 years of follow-up after the second CMR (IQR 2.2–6.9), 40 patients (35%) reached the composite endpoint. Component events were 1 sudden cardiac death, 2 life-threatening ventricular arrhythmias, 6 strokes, 22 episodes of new-onset AFib, and 28 hospitalizations for HF. Among patients with baseline LGE < 15% of LV mass, the relationship between absolute yearly changes in strain and the endpoint is illustrated with spline curves (Fig. 2). In univariable Cox regression, absolute yearly changes in longitudinal strain (*p* = 0.035), radial long-axis strain (*p* = 0.014), and radial short-axis strain (*p* = 0.006) were associated with the endpoint. Conversely, absolute yearly changes in LGE extent were not predictive of outcome (*p* = 0.253). The strongest univariable predictor (absolute yearly change in radial short-axis strain) retained independent prognostic value after adjustment for the HCM risk score and baseline LGE extent (as % LV mass): hazard ratio 1.12 (95% CI 1.03–1.22; *p* = 0.011).

## Discussion

In this multicenter cohort of predominantly low-risk patients with HCM, serial CMR detected subtle but prognostically relevant changes in myocardial structure and function. Over a median of 5.1 years between scans, LV strain parameters worsened in parallel with an increase in LGE burden, supporting a coupled progression of systolic impairment and fibrosis. Importantly, the absolute yearly change in deformation—rather than the single-time-point value—carried prognostic information. After excluding the single patient with significant baseline fibrosis (LGE ≥ 15%), annual worsening in radial short-axis strain remained independently associated with the composite outcome after adjustment for baseline LGE and ESC 5-year SCD risk. Because the ESC HCM risk score was developed to estimate SCD risk (and does not aim to predict new-onset AF or heart-failure hospitalizations), this adjustment should be interpreted as accounting for established SCD risk features rather than implying incremental prediction of SCD itself. In addition, hard arrhythmic endpoints were infrequent and the composite endpoint was dominated by AF and HF admissions; therefore, the prognostic signal observed here most directly pertains to overall clinical morbidity. These observations extend prior studies focused on single-time-point strain or LGE progression by showing that change-based feature-tracking metrics add incremental value over established markers.

The cohort had mild fibrosis at baseline (median LGE 0.5% of LV mass) and largely non-obstructive disease. Although the median age was approximately 60 years—consistent with a clinically early phenotype—disease burden at baseline was low; “low-risk” and “minimally fibrotic” more accurately describe this population than “early-stage.” Despite stable wall thickness and mass, LGE increased to 1.6% at follow-up, highlighting slow but measurable fibrotic progression. In parallel, longitudinal, circumferential, and radial strains deteriorated, indicating evolving myocardial dysfunction even in patients who may appear clinically stable.

A plausible explanation for the stronger signal of radial short-axis strain is that adverse remodeling in HCM—including mid-wall disarray, interstitial fibrosis, impaired energetic reserve, and microvascular dysfunction—preferentially blunts radial thickening. Short-axis feature tracking also benefits from favorable in-plane motion tracking and reproducibility across time, which may sharpen its prognostic performance in serial analyses. Taken together, these factors provide a mechanistic framework for why trajectories in radial thickening track outcomes beyond baseline scar and traditional risk parameters while recognizing that the low frequency of hard arrhythmic endpoints limits inference regarding SCD-specific risk prediction in this cohort.

From a practical standpoint, the findings support an echo-first, CMR-targeted surveillance pathway. Transthoracic echocardiography remains the recommended frontline modality for routine follow-up, and changes in echocardiographic strain (where available) may prompt selective CMR when additional tissue characterization or detailed anatomical assessment is needed. Importantly, echocardiography speckle-tracking strain and CMR feature-tracking strain are not interchangeable: agreement between modalities is only modest and absolute values may differ [15]. Accordingly, strain trends should be interpreted within the context of the imaging modality in which they are measured; echocardiographic strain can serve as a pragmatic trigger for complementary CMR rather than a means to directly translate thresholds or trajectories across modalities. When CMR is obtained serially, tracking radial short-axis strain may help refine clinical risk appraisal—particularly for morbidity outcomes—even in patients with minimal baseline scar.

Several limitations should be acknowledged. The second CMR was performed for clinical reasons rather than on a standardized schedule, introducing selection bias; patients returning for a repeat scan likely had evolving symptoms or clinician concern. This design, together with the requirement for two analyzable CMRs, enriched the cohort for non-obstructive, minimally fibrotic disease and limits generalizability to highly obstructive HCM. The composite endpoint combined heterogeneous outcomes (SCD, ventricular arrhythmias, atrial fibrillation, heart-failure hospitalizations, and stroke), with AF and heart-failure admissions predominating; the models therefore primarily capture predictors of overall morbidity rather than SCD alone. The composite event rate (~ 35%) appears high compared with the median ESC 5-year SCD risk score (2%), a discrepancy likely explained by inclusion of non-SCD morbidity and the tertiary-care setting; it emphasizes that low SCD risk does not equate to low overall morbidity. Median follow-up after the second CMR was 4.3 years, which may not fully capture long-term outcomes in a disease with slow remodeling. Additional limitations include: the retrospective design; potential survivorship and selection biases stemming from retrospectively ascertained serial contrast-CMRs; restricted applicability to obstructive HCM and to patients with higher HCM risk scores; incomplete and inconsistent acquisition of parametric mapping, precluding ECV analysis; and lack of systolic/diastolic strain-rate assessment. The study has also notable strengths including centralized core-lab analysis blinded to clinical data, standardized HCM CMR protocols across centers, LGE quantification by the 6-SD method, and excellent reproducibility of feature-tracking measurements.

In conclusion, in predominantly low-risk, minimally fibrotic HCM, serial CMR reveals subtle declines in LV deformation that track with progressive fibrosis, and annual worsening in radial short-axis strain is associated with subsequent adverse clinical events after the second CMR, independent of baseline LGE and adjusted for ESC 5-year SCD risk.

## Supplementary Information

Below is the link to the electronic supplementary material.

Supplementary Material 1

## Data Availability

Original data are available upon request to the corresponding author.

## Abbreviations

AFib: Atrial fibrillation
CMR: Cardiovascular magnetic resonance
ESC: European Society of Cardiology
HCM: Hypertrophic cardiomyopathy
HF: Heart failure
ICC: Intraclass correlation coefficient
IQR: Interquartile range
LGE: Late gadolinium enhancement
LV: Left ventricular
LVEF: LV ejection fraction
LVMI: Left ventricular mass index
SCD: Sudden cardiac death
