# Supplementary material for: Changes in CMR-derived ventricular strain, fibrosis progression and outcomes in hypertrophic cardiomyopathy
Source: Int J Cardiovasc Imaging. 2026 Jan 27;42(5):893–901. doi: 10.1007/s10554-026-03636-6 (PMC13136199; doi:10.1007/s10554-026-03636-6)
Supplement: Supplementary file 1 — Supplementary Material 1 [file 10554_2026_3636_MOESM1_ESM.docx]

**Supplemental material**

**Changes in CMR-derived ventricular strain, fibrosis progression**

**and outcomes in hypertrophic cardiomyopathy**

Alberto Aimo MD PhD^1,2^, Andrea Barison MD PhD^1,2^, Annamaria Del Franco MD PhD^3^, Chrysanthos Grigoratos MD PhD ^1,2^, Alessandro Parlato MD^4^, Chiara Zocchi MD^3^, Giovanni Donato Aquaro MD PhD^5^, Giorgia Panichella MD^6^, Carmelo De Gori MD^7^, Ignazio Gueli MD PhD ^2^, Alessandro Folgheraiter MD^8^, Antonio De Luca MD^8^, Marco Merlo MD PhD ^8^, Michele Emdin MD PhD ^1,2^, Gianfranco Sinagra MD PhD ^8^, Iacopo Olivotto MD PhD ^3,6,9^, Giancarlo Todiere MD PhD ^2^

1. Interdisciplinary Center for Health Sciences, Scuola Superiore Sant’Anna, Pisa, Italy; 2. Cardiology Division, Fondazione Toscana Gabriele Monasterio, Pisa, Italy; 3. Cardiomyopathy Unit, Careggi University Hospital, Florence, Italy; 4. Cardiology Division, University Hospital of Pisa, Pisa, Italy; 5. Academic Radiology Unit, Department of Surgical Medical and Molecular Pathology and Critical Area, University of Pisa, Pisa, Italy; 6. Department of Experimental and Clinical Medicine, University of Florence, Italy; 7. Radiology Division, Fondazione Toscana Gabriele Monasterio, Pisa, Italy; 8. Cardiology Division, University Hospital of Trieste, Trieste, Italy; 9. Cardiology Unit, Meyer Children’s Hospital IRCCS, Florence, Italy.

**Supplemental Figure 1. Flowchart of patient selection.**


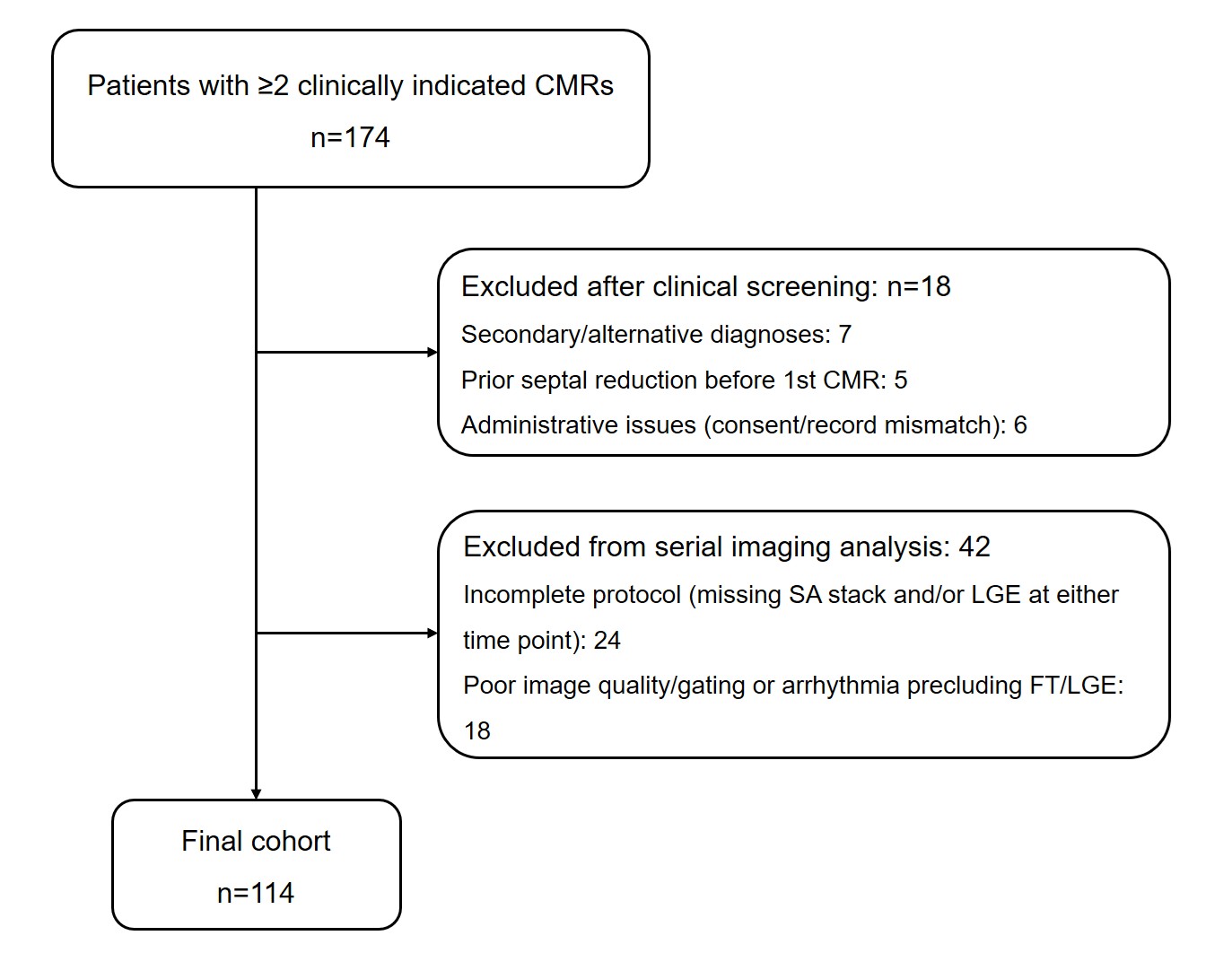


CMR, cardiovascular magnetic resonance; FT, feature-tracking; LGE, late gadolinium enhancement; SA, short-axis.

**Supplemental Table 1. Patient characteristics: obstructive vs. non-obstructive disease.**

|  | **Obstructive HCM**  **n=16 (14%)** | **Non-obstructive HCM**  **n=98 (86%)** | **p** |
| --- | --- | --- | --- |
| Men, n (%) | 12 (75) | 71 (72) | 0.832 |
| Age (years) | 59 (49-69) | 49 (35-59) | **0.018** |
| Palpitations, n (%) | 6 (38) | 29 (30) | 0.525 |
| Unexplained syncope, n (%) | 1 (6) | 5 (5) | 0.849 |
| Angina, n (%) | 6 (38) | 19 (19) | 0.104 |
| Dyspnea on effort, n (%) | 10 (63) | 26 (27) | **0.004** |
| Atrial fibrillation, n (%) | 3 (19) | 16 (16) | 0.809 |
| NSVT, n (%)* | 3 (25) | 11 (16) | 0.444 |
| Family history of SCD, n (%)* | 1 (8) | 11 (16) | 0.514 |
| LVOT gradient (mmHg) | 15 (3-46) | 5 (2-10) | **0.028** |
| Maximal LV wall thickness (mm) | 20 (17-22) | 17 (15-19) | **0.012** |
| Left atrial diameter (mm) | 41 (35-44) | 38 (34-43) | 0.530 |
| HCM score (%) | 2 (2-3) | 2 (2-3) | 0.767 |

* Percentage calculated out of the available data. Significant p values are reported in bold. HCM, hypertrophic cardiomyopathy; LV, left ventricle; LVOT, left ventricular outflow tract; NSVT, non-sustained ventricular tachycardia; NYHA, New York Heart Association; SCD, sudden cardiac death.

**Supplemental Table 2. Changes in left ventricular strain values.**

|  | Absolute change | Absolute change per year |
| --- | --- | --- |
| Longitudinal (%) | +1 (-1/+3) | 0 (0/+1) |
| Circumferential (%) | 0 (-1/+2) | 0 (0/0) |
| Radial long-axis (%) | -2 (-6/+2) | 0 (-1/0) |
| Radial short-axis (%) | 0 (-4/+3) | 0 (-1/+1) |
